# “All Bookshelves Are Magical”

**DOI:** 10.3201/eid2610.AC2610

**Published:** 2020-10

**Authors:** Byron Breedlove

**Affiliations:** Centers for Disease Control and Prevention, Atlanta, Georgia, USA

**Keywords:** art science connection, emerging infectious diseases, art and medicine, about the cover, all bookshelves are magical, Yi Taek-gyun, books and scholars’ accouterments, books, bookcases, Chaekgeori, Chosŏn Dynasty, social distancing, quarantine

**Figure:**
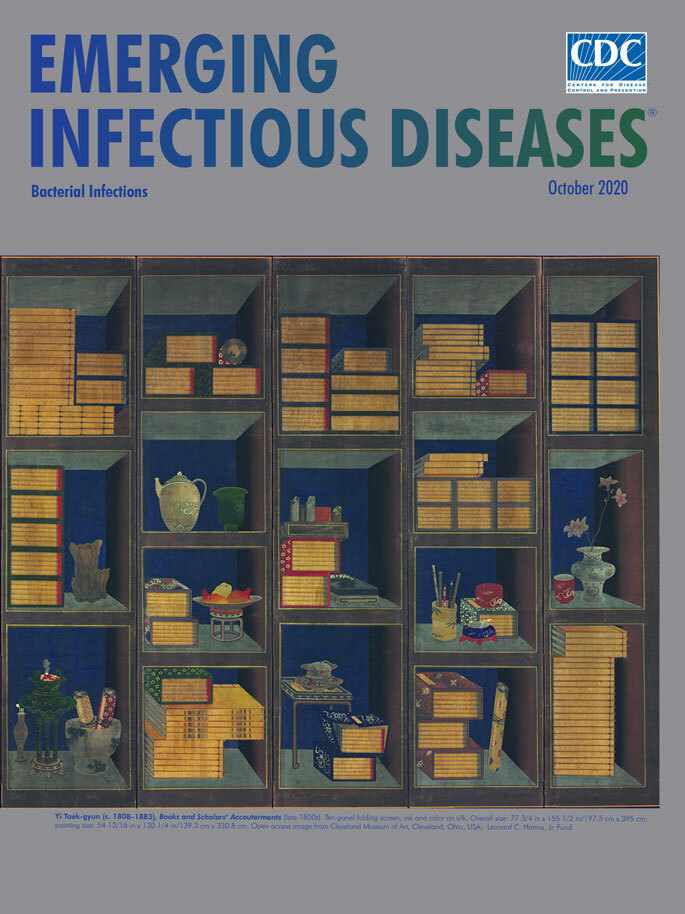
**Yi Taek-gyun (c. 1808–1883), Books and Scholars’ Accouterments (late 1800s)**. Ten-panel folding screen; ink and color on silk. Overall size: 77 3/4 in × 155 1/2 in/197.5 cm × 395 cm; painting size: 54 13/16 in × 130 1/4 in/139.3 cm × 330.8 cm. Open access image from The Cleveland Museum of Art, Cleveland, Ohio, USA; Leonard C. Hanna, Jr. Fund.

During these times when social distancing and quarantining are widely practiced, people around the world are watching news or entertainment being broadcast from makeshift home studios and teleconferencing to stay connected with staff, team members, collaborators, family, and friends. Frequently sharing screen time with the speakers are all manner of bookshelves in the background, and the collections of books and ephemera on the shelves have provided grist for stories and commentaries by many journalists and bloggers throughout the year. 

For instance, Vogue.com editor Stuart Emmrich admits to becoming obsessed with what is in the background—especially the books: “Ah, yes, bookshelves. Rows of carefully arranged books seem to be the go-to choice of most of the reporters and commentators who provide the bulk of the cable-news programming. Thus, my curiosity about their reading habits.” For those of us without our own curated collections of books to share, photographs of shelves brimming with books are available as virtual stand-ins. Penguin Random House even has images of “credibility bookshelf” backgrounds available to download. 

Featuring bookcases in the background is not, however, a novel idea by any means. King Jeongjo, the 22nd ruler of the Korean Chosŏn (also called Yi) Dynasty during 1776–1800, was an early proponent of this practice. He positioned a painted screen displaying books and other objects behind his throne. Art historian Sunglim Kim explains that the king used the screen “as a vicarious substitute for reading and studying, as he did not have as much time to spend with his books as he wanted.”

Known as *Chaekgeori,* this style of still life painting flourished during the latter part of the Chosŏn Dynasty, the last and longest-lived imperial dynasty (1392–1910) of Korea*.* Sooa McCormick, Assistant Curator of Korean Art, Cleveland Museum of Art, notes that *Chaekgeori* is translated into English as “books and things.” Works in this genre reflect an admiration for learning and scholarship, and effects akin to those found in Western *trompe l’oeil* (French: deceive the eye) painting were commonly used to create the three-dimensional spatial illusion characteristic of these compositions. 

Most *Chaekgeori* are not signed or dated; consequently, the identities of many of their creators remain unknown. *Books and Scholars’ Accouterments*, this month’s cover image, is a rare exception. The Cleveland Museum of Art explains that the third panel from the right features a hidden seal that reveals the artist as Yi Taek-gyun. To date, only about a dozen such hidden seal impressions have been found, including three for this artist. Despite his standing as an established court artist, details about the life and work of Yi Taek-gyun are scarce. The Asian Art Museum, San Francisco, notes that he came from a family of court painters and that he changed his name several times. He used Yi Hyeongrok until 1864 and Yi Eungrok from 1864 to 1871 before switching to Yi Taek-gyun.

Extending across 10 folding panels, *Books and Scholars’ Accouterments* depicts unusual and luxurious accessories that a 19th-century Korean scholar may have collected and displayed in a private study. Viewed as a montage, this tableau is dominated by a uniformly dark blue background, neatly stacked books with honey-colored pages, and objects carefully arranged on the shelves. The orthogonal lines that define the shelves and the contrasting dark shading for the background and light shading for the tops and bottoms of the shelves create a perception of recessed space and consistent depth. 

Books, the primary motif within this still life genre, appear on 27 shelves*.* Some alcoves hold only books; others also feature writing implements, ceramics, pottery, flowers, and exotic luxuries and delicacies. Specifically among the myriad items showcased are peacock feathers, a bamboo brush holder, a three-tier lunch container, a red cup and lid, a thin crackle-patterned vase, narcissus flowers, scrolls jutting from a translucent glass bowl, a red incense burner on a tripod, and a plate of pomegranates and finger citrons on a wooden stand. 

Yi Taek-gyun’s mastery of colors, textures, and details is apparent. Kim explains that the challenge of creating diverse collections of items was appealing for *Chaekgeori* painters who “explored every visual possibility of the object—shape, color, and texture—to create a feast of sensuality.” 

Contemporary English author Neil Gaiman once said, “All bookshelves are magical.” Indeed, *Chaekgeori* paintings reveal something of the wonder and joy of books, their historical association with knowledge and scholarship, and even the dynamic struggle between order and chaos often playing out on our bookshelves.

Since the French *Journal des Sçavans* and the English *Philosophical Transactions of the Royal Society* debuted in 1665, book reviews have been staples of scientific and scholarly periodicals. *Emerging Infectious Diseases* published its first book review in September 1997. Including that review for *Virus Hunter* from 1997 and the one for *The Mosquito: Human History of Our Deadliest Predator* appearing in this issue, the journal has published 236 book reviews that cover an assortment of subjects apropos to understanding factors involved in disease emergence, prevention, and elimination.^1^ Readers of this journal no doubt have many of those books in their own collections and perhaps can enjoy envisioning what a *Chaekgeori* painting featuring their own books and scholarly accouterments would include.

## Footnotes

1 Readers searching for specific books reviewed in *Emerging Infectious Diseases* have a pair of options available: examine the list of books and media reviews by year or use the article index by types search feature.
